# Analysis of the movement of permanent GNSS stations in Spain with directional statistics

**DOI:** 10.1038/s41598-026-39548-7

**Published:** 2026-04-03

**Authors:** Fernando Broncano, Aurora Cuartero, Pablo G. Rodríguez, Antonio Plaza

**Affiliations:** 1 Department of Computer and Telematic Systems Engineering, School of Industrial Engineering, University of Extremadura, Av. de Elvas, s/n, Badajoz, 06006 Extremadura Spain; 2 Department of Graphic Expression, School of Technology, University of Extremadura, Av. de la Universidad, s/n, Cáceres, 10003 Extremadura Spain; 3 Department of Computer and Telematic Systems Engineering, School of Technology, University of Extremadura, Av. de la Universidad, s/n, Cáceres, 10003 Extremadura Spain; 4 Department of Computer and Communications Technologies, School of Technology, University of Extremadura, Av. de la Universidad, s/n, Cáceres, 10003 Extremadura Spain

**Keywords:** GNSS, EPN, PyCircularStats, Circular statistics, Tectonic plate movements, Engineering, Mathematics and computing

## Abstract

Global Navigation Satellite System (GNSS), such as Galileo, GPS, GLONASS, and BeiDou, provide essential global positioning, navigation, and timing data used across a wide range of scientific and engineering applications. These systems enable precise monitoring of civil infrastructure, crustal deformations, and geodetic reference frame updates. In particular, the European Reference Frame (EUREF) Permanent Network (EPN) offers a long-term dataset of GNSS measurements valuable for multi-disciplinary analyses. This paper explores the use of circular statistics to analyze directional data derived from EPN stations distributed across Spain. Our study provides both numerical and graphical representations of GNSS vector data to uncover temporal trends and spatial patterns in station movements. The visual interpretation of directional data enhances the understanding of GNSS-derived displacement behavior. This work aims to make such analyses more accessible by promoting intuitive, graphical tools over traditional, often proprietary statistical software, thereby supporting broader engagement in GNSS-based geospatial research. Among the findings, the study reveals that all permanent stations exhibit a northeastward shift of $69.27\mu m/d$ (2.53*cm*/*y*) with an angle of $41.21^\circ$, approximately 1 mm every 14 days.

## Introduction

Global Navigation Satellite System (GNSS)^1^ is a positioning technology supported by a constellation of satellites that provides global coverage positioning and timing data to GNSS receivers. The receivers use these data to determine their location. Examples of GNSS include Galileo (the European Global Navigation Satellite System), NAVSTAR-GPS (the USA Navigation System Using Time and Ranging-Global Positioning System), GLONASS (the Russian Global’naya Navigatsionnaya Sputnikovaya Sistema) and BeiDou (the Chinese Navigation Satellite System).

A GNSS provides essential support for multiple positioning, navigation, and timing applications. GNSS is already the foundation of performance-based navigation, and it provides a common time reference that is used to synchronize aviation systems, communication networks and operations, as well as a wide range of positioning-related applications^2–10^.

Different examples of important analytics based on GNSS measures can be found in the literature, including civil structure monitoring, geological phenomena such as the crustal movements and local displacements, geodetic coordinate frame updating, and even the monitoring of GNSS metadata and data validation in networks in which these techniques are supported. As an example of the above analytics, in^11^ a data processing framework was presented that takes advantage of GNSS measurements to analyze and predict the movements of bridges. The proposed methodology allows the estimation of its movements using the Kalman filter and neural network techniques. Besides, it enables the comparison of the dominant frequencies of the least squares harmonics estimation of bridge movements with those of the permanent stations, to provide information on the stability of civil structures. Another example is given in^12^, where GNSS data are used for the monitoring of the deformations of the Earth’s crust. Three case studies of crustal deformations were analyzed: 1) in the Indian Ocean, the Sumatra-Andaman earthquake occurred in 2004; 2) in Japan, Geographical Survey Institute (GSI) established global positioning system (GPS)-based control stations throughout the country; 3) In the Alps, with two different geodetic GNSS networks. Conclusions showed that these types of analytics based on GNSS data allowed for the development of more realistic geophysical models, whereas the main difficulty is still how to verify the reliability of the estimated deformations, and to explain their physical causes (tectonic drift, seasonal effects, etc.). Cheng *et al.*^13^ also introduced an optimal method to align the Chinese regional network to an International Terrestrial Reference Frame (ITRF) and to update coordinates of the stations of the China Geodetic Coordinate System 2000 (CGCS2000) considering the movement of the Earth plates.

The European Reference Frame (EUREF) Permanent Network (EPN) is a network of GNSS stations in continuous operation installed throughout the European continent. GNSS metadata and data validation in the Permanent EUREF Network have been presented in^14,15^. The EPN Central Bureau (CB) performs a daily coordination of the EPN, through effective communication between operators, stations, data centers and analysis centers. In addition, the CB maintains the EPN incorporating the required improvements, such as the new general data protection regulation, new multi-GNSS signals, new receiver independent exchange (RINEX) formats, thus allowing greater use of GNSS data in real time. The analysis of 23 years of quality controls of EPN GNSS data can be found in^15^. Thanks to numerous national and international collaborations, the number of permanent GNSS stations and monitoring networks is constantly growing, increasing the possibilities of studies based on these GNSS systems.

Circular data, as part of directional data, are used in a wide range of fields. Analyzing directional data can be useful in different earth science and astrophysical areas to analyze movements, based for example on the GNSS data previously described.

Despite some variations in the context, the methodology used for statistical analysis is roughly the same in most situations. Currently, there is a plethora of datasets publicly available which can be used in numerous applications and in different lines of research. The datasets from the permanent network station described above are only one representative example. However, modern automatic data processing tools are needed to perform such statistical analysis tasks efficiently and effectively. For this reason, in this work we adopt tools that enable the graphic interpretation of some circular statistics calculated for improved data analysis.

Currently, the analysis of data (and, specifically, its representation) is performed with specialized software, which is usually proprietary, and it is developed using platforms and languages that are not very inclusive for professionals that find their background far from the statistics domain. Examples of these tools are SPSS, XLSTATS, MATLAB and R. An example is the “CircStat” toolbox in Matlab^16^.

In this paper, we use circular statistics (such as those available in a previously developed open-source library implemented to be executed by the Python interpreter, called PyCircularStats^17^) to perform a multi-temporal analysis using the locations of a number of permanent stations of the EPN. In this case study, the results calculated with numerical circular statistics were compared with the graphic results. The possibility of visualization of the vectors with their orientations and modules allows for a more intuitive analysis of the obtained results. Furthermore, these results allow us to study the trend of the movement of the analyzed stations. For this purpose, all permanent stations of the EUREF network located within Spanish territory have been selected. These stations define the study area of this work. The aim is to analyze the long-term movement trends in Spain and observe their variation over time.

The rest of the paper is organized as follows. Section "Fundamentals on circular statistics" describes previous works related to circular data. Section "Materials and methods" describes the materials and methods used in this study. A use case focused on the analysis of the locations of the Spanish EPN stations is discussed in Section “Case study”. Finally, some conclusions are given in Section “Conclusions”.

## Fundamentals on circular statistics

In the following section, various state-of-the-art works on circular statistics and their application to different cases of movement analysis is explored.

### Circular statistics

Circular statistics provide a methodological framework for analyzing directional data, angles, orientations, or axial measurements, that exhibit periodicity and lack a natural origin. Unlike linear variables, circular observations lie on the unit circle and wrap around at $2\pi$ (or $360^\circ$), which requires adapted definitions of central tendency, dispersion, and distributional modeling. A commonly used probability model for uni-modal circular data is the von Mises distribution^18^, whose probability density function is

1 $$\begin{aligned} p(x, \delta _1, \kappa ) = \frac{1}{2\pi I_0(K)} \cdot e^{\left( \kappa \cdot \cos (x - \delta _1)\right) } \end{aligned}$$

where $I_0(K)$ is the modified Bessel function of the first kind and order zero, *K* is the concentration parameter (analogous to precision in linear statistics), and $\delta _1$ is the mean direction.

For a set of azimuths $\{\alpha _1, \dots , \alpha _N\}$, the mean direction is computed from the average cosine and sine components:

2 $$\begin{aligned} \left( \sum _{i=1}^N \cos \alpha _i,\; \sum _{i=1}^N \sin \alpha _i \right) \end{aligned}$$

The mean resultant length,

3 $$\begin{aligned} \tilde{R} = \frac{R}{N} \qquad R = \sqrt{\left( \sum _{i=1}^N \cos \alpha _i\right) ^2 + \left( \sum _{i=1}^N \sin \alpha _i\right) ^2} \end{aligned}$$

quantifies the concentration of the observations around the circle. From $\tilde{R}$, the circular variance (*V*) and circular standard deviation (*s*) follow as

4 $$\begin{aligned} V= & 1 - \tilde{R} \end{aligned}$$

5 $$\begin{aligned} s= & \sqrt{-2 \ln (\tilde{R})} \end{aligned}$$

Higher-order descriptors include circular skewness (*b*) and kurtosis (*k*), which measure asymmetry and shape relative to the mean direction $\bar{\alpha }$:

6 $$\begin{aligned} b = \frac{1}{N} \sum _{i=1}^{N} \sin \left[ 2(\alpha _i - \bar{\alpha })\right] \end{aligned}$$

7 $$\begin{aligned} k = \frac{1}{N} \sum _{i=1}^{N} \cos \left[ 2(\alpha _i - \bar{\alpha })\right] \end{aligned}$$

When observations correspond to directional vectors rather than pure angles, the mean vector magnitude is

8 $$\begin{aligned} \bar{X} = \frac{1}{N} \sum _{i=1}^{N} \sqrt{(x_{2i}-x_{1i})^2 + (y_{2i}-y_{1i})^2} \end{aligned}$$

allowing simultaneous analysis of orientation and length. These methods together provide a complete statistical framework for evaluating directional patterns, concentration, dispersion, and structural organization in circular datasets.

A variety of software tools support circular statistical analysis. The VecStatGraphs2D package for R^19^ provides extensive graphical and descriptive functionality, although it is limited in terms of API availability for automated processing. To address this, the present work employs PyCircularStats, a Python library designed for analysing two-dimensional directional vectors of arbitrary magnitude^17^. This package complements VecStatGraphs2D by providing both circular and non-circular representations of directional datasets. Additionally, the QCircularStats plugin for QGIS^20^ offers an accessible geospatial interface for circular analysis. For a comprehensive overview of theoretical foundations, we refer the reader to standard texts on circular and directional statistics^21,22^.

### Applied cases of movement analysis

Recent studies have investigated plate motion using GNSS data. The following are some notable examples. In^23^, tectonic movements in northeast Japan between 2000 and 2022 are analyzed using GNSS data and tide gauge measurements. The impact of the 2011 Tohoku-Oki earthquake and eight other seismic events, which caused interruptions in the GNSS time series, is highlighted. Velocity vectors show significant internal variations, especially in the central and southern regions, suggesting deformation related to distinct geological terrains.

^24^ compares the point velocities using global reference frames (IGS) and regional reference frames (ETRF). For Poland’s land cadastre, the accuracy of the boundary point must be better than 0.30m. Over 25 years, tectonic plate movement can cause shifts of up to 0.13 m, which is still within acceptable limits.

The study in^25^ employed Precise Point Positioning (PPP) techniques and used data from Continuously Operating Reference Stations (CORS) in Vietnam from 2016 to 2018 to estimate tectonic motion velocities in Ha Noi, Da Nang, and Ho Chi Minh City. The results indicated that the velocities of tectonic movement in the North, East, and Up components were (−13.1, +32.8, −1.3) mm/year in Ha Noi, (−9.9, +31.0, +2.6) mm/year in Da Nang, and (−10.3, +26.9, +2.7) mm/year in Ho Chi Minh City.

## Materials and methods

This section describes the materials and methods used to carry out this work. First, subsection "Provided data from stations" provides a description of the data used from permanent stations, and subsection “Directional analysis” describes the methodology that will be applied to the positions. Similarly, subsections “Programming language” and “External libraries” describe the programming language and support libraries required for graphical representation and numerical analysis, respectively.

### Provided data from stations

**Fig. 1:**
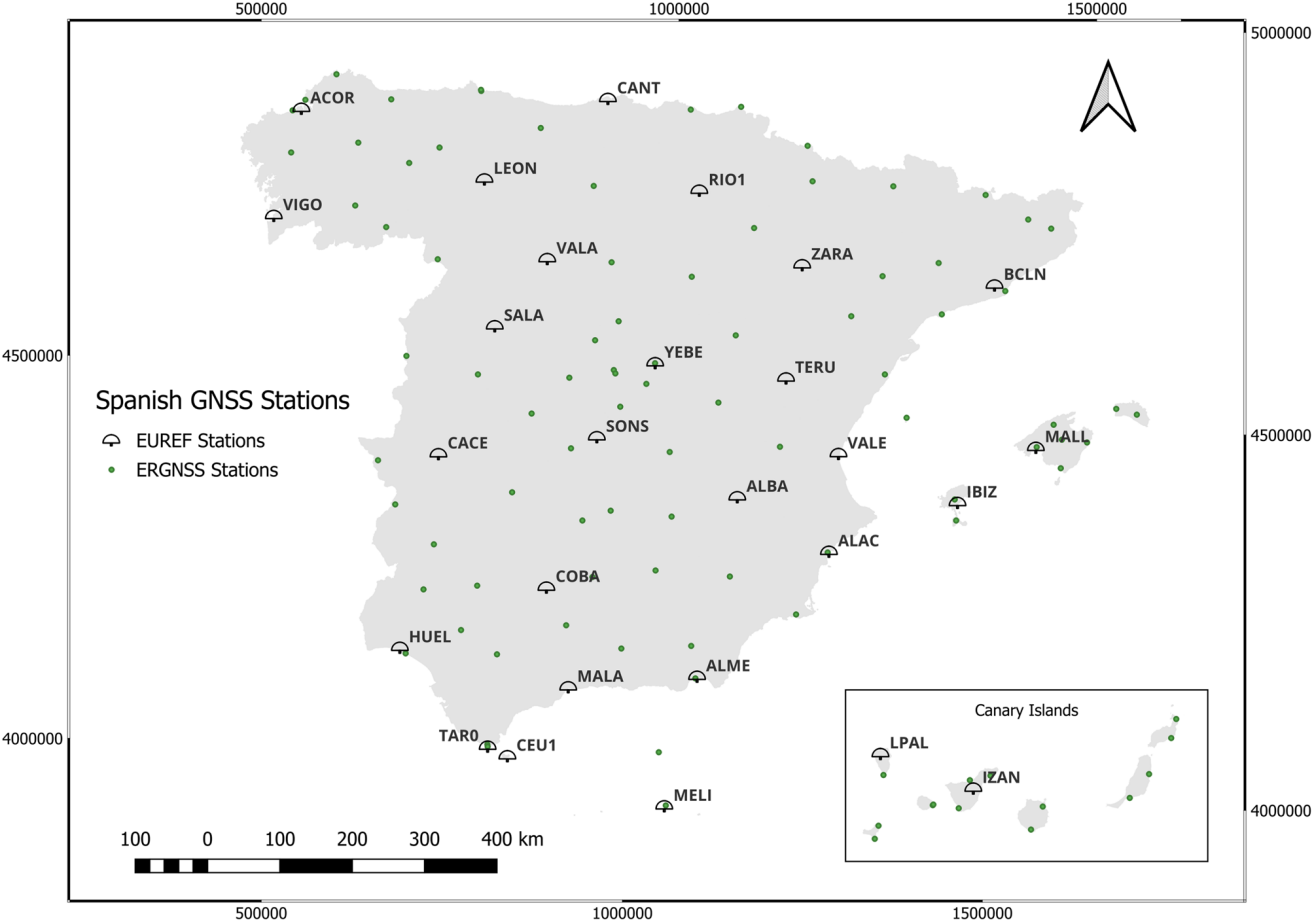
Geographic location of Spanish permanent GNSS Stations Network (ERGNSS). One hundred twenty-four of them are permanent stations, and twenty-seven of these belong to the EUREF EPN. Map generated with QGIS 3.34 LTR^26^.

The data used for this study comes from the Spanish EPN and are shown in Fig. 1 and Table 1. In 1998, the Spanish National Geographic Institute (IGN), started to install the national GNSS Permanent Stations Network, with the first station installed in the tide gauge of Alicante, vertical *datum* for the Spanish Height System. This station was the first integrated in the EPN (ALACOOESP). Later, new stations were installed mainly in other observatories and tide gauges, like YEBEOOESP, co-located next to the former 14 m VLBI antenna of the Yebes Observatory. The main objectives of the network were:

1. To contribute to the realizations of International Terrestrial Reference Frame (ITRF) and European Terrestrial Reference System 1989 (ETRS89) in Spain.
2. To provide public data for users of cartography, civil engineering, navigation, earth sciences investigation, etc.
3. To constitute an active network which provides access to ETRS89 through real time positioning.

**Table 1:** Summary of data from EUREF permanent stations analysed in this study.

Station	City and Province	Initial Date	Final Date	Days	Years	N
ACOR	A Coruña, A Coruña	1998 Dec, 11	2024 Sep, 28	9423	25.8	8829
ALAC	Alicante, Alicante	1998 Mar, 06	2024 Sep, 28	9703	26.6	9333
ALBA	Albacete, Albacete	2002 Jul, 10	2024 Sep, 28	8116	22.2	7990
ALME	Almería, Almería	1999 Dec, 04	2024 Sep, 28	9065	24.8	8739
BCLN	Sant Vicenç dels Horts, Barcelona	2012 Feb, 07	2024 Sep, 28	4617	12.6	4525
CACE	Cáceres, Cáceres	2000 Dec, 05	2024 Sep, 28	8698	23.8	8387
CANT	Santander, Cantabria	2000 Mar, 12	2024 Sep, 28	8966	24.6	8763
CEU1	Ceuta, Ceuta	2007 Aug, 25	2024 Sep, 28	6244	17.1	6088
COBA	Córdoba, Córdoba	2004 Apr, 14	2024 Sep, 28	7472	20.5	7134
HUEL	Palos de la Frontera, Huelva	2003 Apr, 14	2024 Sep, 28	7838	21.5	7466
IBIZ	Elvissa, Ibiza	2004 Oct, 14	2024 Sep, 28	7289	20.0	6189
IZAN	Güímar, Tenerife	2008 May, 16	2024 Sep, 28	5979	16.4	5897
LEON	León, León	2007 Mar, 25	2024 Sep, 28	6397	17.5	6340
LPAL	Garafía, La	2001 May, 10	2024 Sep, 21	8535	23.4	8408
MALA	Málaga, Málaga	2000 May, 05	2024 Sep, 28	8912	24.4	8788
MALL	Palma, Mallorca	2000 May, 12	2024 Sep, 28	8905	24.4	8749
MELI	Melilla, Melilla	2011 Nov, 24	2024 Sep, 28	4692	12.9	4533
RIO1	Logroño, La Rioja	2012 Jan, 31	2024 Sep, 28	4624	12.7	4563
SALA	Encinas de Abajo, Salamanca	2006 Jun, 29	2024 Sep, 28	6666	18.3	6519
SONS	Sonseca, Toledo	2000 Nov, 25	2024 Sep, 28	8708	23.9	8597
TAR0	Tarifa, Cádiz	2019 Jun, 06	2024 Sep, 28	1941	5.3	1841
TERU	Teruel, Teruel	2008 Mar, 07	2024 Sep, 28	6049	16.6	5761
VALA	Valladolid, Valladolid	2007 Nov, 13	2024 Sep, 28	6164	16.9	6125
VALE	Valencia, Valencia	2000 Jan, 10	2024 Sep, 28	9028	24.7	8529
VIGO	Vigo, Pontevedra	2001 Sep, 12	2024 Sep, 21	8410	23.0	8260
YEBE	Yebes, Guadalajara	1999 May, 19	2024 Sep, 21	9257	25.4	9000
ZARA	Zaragoza, Zaragoza	2006 Apr, 18	2024 Sep, 28	6738	18.5	6646

Currently, the Spanish EPN is made up of 124 permanent GNSS stations that are distributed throughout the national territory. Of the 124 stations, 27 are integrated into the European network of EUREF, and 6 are part of the global network of the IGS (International GNSS Service). The coordinates of these stations are calculated using precision scientific software (coordinates of ERGNSS stations) as BERNESE^27^.

In this study, we have selected the Spanish stations that belong to the EUREF network (see Fig. 1). This set of stations has been working long enough to provide data that are significant for multitemporal analysis.

Table 1 shows all current permanent stations in Spain and their operational period. In addition, this table and Fig. 1 show the permanent stations selected to perform the data analysis. The criteria used to select these stations was guided by the longest possible time and a homogeneous distribution across Spain.

In Table 1, the “Station” column shows the name of the station without the “00ESP” suffix, and together with the “Place” column, it indicates its location on the political map of Spain. The table also shows the start and end dates of the position measurements. These intervals are presented in days, years, and numbers of values analyzed (N). The station TAR0 has a particular feature, as it changed from TARI to TAR0 in 2019 following a sudden position shift (approximately 8 meters). Therefore, all measurements taken before June 6, 2019, for TARI are not valid.

Once the data to be used are defined, they are obtained from the permanent station data provided by the University of Nevada, which has been processed and made publicly available. These data have been processed using BERNESE for the generation of daily positions. To obtain accurate calculations, it is necessary to recompute the ephemerides over a large time interval^28^. The available data include the position of each permanent station for every day from its installation up to the end date, as shown in Table 1. The data source is available at the following link: https://geodesy.unr.edu/^29^.

### Directional analysis

The data are provided in the WGS84 reference system as latitude and longitude. These positions have been converted to the Mercator UTM30 projection, the zone used in Spain. These conversions were performed using the PyProj library for Python. In this way, a study of circular coordinates can be carried out using the UTM reference system, as these positions are considered within a Cartesian plane.

After converting the positions to the new reference system, daily movements can be analyzed to estimate displacement. Daily movements are calculated as the difference in position between day *i* and day $i+1$, according to:

9 $$\begin{aligned} \Delta p = p_{i+1} - p_i \end{aligned}$$

where $p = \{ X_{\text {UTM30}},\ Y_{\text {UTM30}} \}$. Each dataset of positions from each station is transferred to a file with four columns, two for each component of $p_i$ and $p_{i-1}$. Once the movements for each station are obtained, they are processed using PyCircularStats. This software provides a file containing the modulus, angle, increment in the X component, and increment in the Y component, along with the input position data. These data are used to provide the resulting motion and displacement vectors for each station.

The results were calculated following three strategies:

- Strategy 1: Each of the $\Delta p$ values is summed, resulting in a vector that represents the total of daily movement increments. This vector is obtained by summing each movement component day by day. This strategy provides the displacement of the station over the entire measurement interval.
- Strategy 2: The average position of the first fifteen days and the last fifteen days is computed for each station, and the movement between these two average positions is calculated. Since this movement spans a period of more than five years, it can be considered trend-based, and therefore, it is interpreted as displacement.
- Strategy 3: The final strategy calculates the average of the daily increments for each station. The displacement derived from the first strategy is divided by the number of processed values to obtain an average that should remain linearly independent of the number of measurements and the overall displacement. The angle of this strategy is the same as that of Strategy 1.

### Programming language

Python is the language used in this work due to the facilities it offers in terms of libraries and external frameworks for the processing of mathematical operations and their graphical representation. In addition, its performance is usually better than that of R^30^, mainly due to the multi-threaded nature of Python. Our implementation was carried out using version 3 of Python. In this work, a virtual environment in Python 3.10 has been employed.

### External libraries

Several libraries have been utilized for the analysis of positions, their conversion between different reference systems, mathematical operations involving these data, and their graphical representation on a plane.

NumPy^31^ is a Python library used for scientific calculation that simplifies code maintenance, allowing to integrate basic algebraic operations in very simple and easy-to-use manner. In addition, it parallelizes array operations, using different threads for each value contained in this structure, which represents a great performance increase with respect to R. The use of this library has been essential to meet the performance objectives required during the analysis of large datasets. On the other hand, Scipy^32^ is a library used for scientific and algebraic calculation that complements with its functions some algorithms that are not available in NumPy.

For the graphical representation of data, MatPlotLib^33^ is employed. It allows to represent (in a very simple way) bi-dimensional and tri-dimensional scenes, including different objects such as vectors, curves, surfaces, etc. It also allows navigation in the representation, including zoom, rotations, and translations. This library also enables exporting an image of the scene in high resolution, which is useful for users of the library to print the results of their research, being able to share them with the rest of the community in any graphics format.

The transformations between the different positional reference systems are performed using the PyProj^34^ library for Python. It allows transforming geographic coordinate pairs (latitude/longitude) into projected Cartesian coordinates, such as the Mercator reference system. This is useful for processing datasets across different terrestrial reference systems.

Finally, The PyCircularStats^17^ library implements both types of statistics: linear and circular. The linear statistics methods are used to analyze the module or magnitude of the vectors. The implemented methods provide descriptive statistics, including the number of elements, the arithmetic mean, the maximum and minimum values, the population and the sample variances and deviations, standard error, the coefficient Skewness for establishing the level of asymmetry of the probability distribution and the Kurtosis measure to calculate how flat or steep is the distribution.

## Case study

This section presents the results of the analysis carried out on the positions of the permanent stations. Table 2 presents the results calculated using the three strategies, obtaining both the magnitude and angle for the position increments as $\Delta {x}$ and $\Delta {y}$ both through summation and by analyzing the movement between the first fifteen and the last fifteen measurements as $\widehat{\Delta {x}}$ and $\widehat{\Delta {y}}$. Strategies 1 and 2 express displacement vectors in meters (m) and angles in degrees ($\phantom{0}^{\circ }$), representing cumulative displacement and providing complementary assessments of long-term secular tectonic velocity. Strategy 3, in contrast, presents the mean daily displacement magnitude, computed by dividing Strategy 1 results by the number of available observation values (N), which differs from the total number of calendar days in the observation period due to missing daily measurements caused by data gaps or discontinuities. The directional angle remains identical to Strategy 1, as scalar division does not alter vector direction. Thus, while Strategies 1 and 2 characterize multi-year tectonic trends, Strategy 3 quantifies the average daily displacement rate based on available observations.

**Table 2:** Displacement results for the three strategies analyzed. Increments and modules are shown in meters (*m*), and angles in degrees $({}^\circ )$.

Station	Values	**Strategy 1**				**Strategy 2**				**Strategy 3**
		$\Delta {x}$	$\Delta {y}$	Module	Angle	$\widehat{\Delta {x}}$	$\widehat{\Delta {y}}$	Module	Angle	$\frac{Module}{Values} \ (\mu {m}/N)$
ACOR	8829	0.6189	0.4033	0.7387	33.09	0.6147	0.4042	0.7357	33.32	83.6676
ALAC	9333	0.5135	0.4531	0.6848	41.43	0.5147	0.4531	0.6857	41.36	73.3788
ALBA	7990	0.4036	0.3700	0.5475	42.51	0.4039	0.3682	0.5465	42.35	68.5232
ALME	8739	0.4579	0.4005	0.6083	41.18	0.4559	0.4037	0.6090	41.52	69.6096
BCLN	4525	0.2381	0.2126	0.3192	41.76	0.2394	0.2143	0.3213	41.83	70.5368
CACE	8387	0.4482	0.3834	0.5898	40.55	0.4492	0.3835	0.5907	40.48	70.3283
CANT	8763	0.4528	0.4092	0.6103	42.10	0.4523	0.4050	0.6071	41.83	69.6463
CEU1	6088	0.2749	0.2850	0.3959	46.03	0.2740	0.2829	0.3938	45.91	65.0366
COBA	7134	0.3813	0.3219	0.4990	40.17	0.3837	0.3216	0.5007	39.97	69.9484
HUEL	7466	0.3984	0.3486	0.5294	41.18	0.3974	0.3499	0.5295	41.36	70.9094
IBIZ	6189	0.3897	0.3385	0.5162	40.98	0.3912	0.3403	0.5185	41.02	83.4034
IZAN	5897	0.2879	0.2462	0.3788	40.53	0.2843	0.2478	0.3771	41.06	64.2432
LEON	6340	0.3242	0.2742	0.4246	40.22	0.3221	0.2761	0.4242	40.60	66.9722
LPAL	8408	0.4351	0.3457	0.5557	38.46	0.4332	0.3451	0.5539	38.54	66.0951
MALA	8788	0.4288	0.3609	0.5604	40.09	0.4269	0.3606	0.5589	40.18	63.7735
MALL	8749	0.4715	0.4286	0.6371	42.27	0.4722	0.4263	0.6361	42.07	72.8228
MELI	4533	0.2234	0.2368	0.3255	46.66	0.2222	0.2385	0.3260	47.03	71.8157
RIO1	4563	0.2336	0.2013	0.3084	40.75	0.2335	0.2048	0.3106	41.25	67.5782
SALA	6519	0.3456	0.2861	0.4487	39.62	0.3435	0.2883	0.4485	40.00	68.8292
SONS	8597	0.4652	0.3916	0.6081	40.09	0.4612	0.3892	0.6035	40.16	70.7352
TAR0	1841	0.0428	0.1964	0.2010	77.70	0.0413	0.1921	0.1965	77.87	109.1703
TERU	5761	0.3152	0.2734	0.4173	40.94	0.3165	0.2729	0.4179	40.76	72.4289
VALA	6125	0.3179	0.2705	0.4174	40.39	0.3160	0.2698	0.4155	40.49	68.1391
VALE	8529	0.4753	0.4184	0.6332	41.36	0.4722	0.4170	0.6299	41.45	74.2437
VIGO	8260	0.4348	0.3565	0.5622	39.35	0.4320	0.3563	0.5600	39.51	68.0653
YEBE	9000	0.4824	0.4110	0.6337	40.43	0.4774	0.4096	0.6290	40.62	70.4128
ZARA	6646	0.3443	0.2986	0.4557	40.93	0.3434	0.3021	0.4574	41.33	68.5728

All the positions of all the stations used to compute the averages for the first and last 15-days fall within the interval $[\mu - 3\sigma ,\ \mu + 3\sigma ]$, providing statistical significance. The 15-day window was selected to balance statistical robustness with data efficiency. This window size is sufficiently large to provide meaningful positional averages while remaining small enough to avoid convergence effects in stations with shorter time series. Specifically, the 15-day window represents approximately 1.5% or less of the total available data for each station, with the limiting case being station TAR0 (1,841 measurements). This ensures that the displacement estimates are representative of the endpoints of the time series without being unduly influenced by extended temporal averaging.

Additionally, we verified that all measurements within the initial and final 15-day windows were free from documented discontinuities based on IGN station logs (Station logs and discontinuity information are publicly available from the Instituto Geográfico Nacional (IGN) geodesy data repository. The quality control files can be accessed at: https://datos-geodesia.ign.es/ERGNSS/diario_30s/YYYY/, where YYYY corresponds to the year of interest. The relevant file follows the naming convention CHECK_IMPORT*.ergnss, where the asterisk may include additional year or version identifiers). In three isolated cases (two at the initial epoch and one at the final epoch, across three different stations), individual daily positions fell outside the $[\mu - 3\sigma ,\ \mu + 3\sigma ]$ interval and were excluded, with the next (or previous) available day substituted to maintain the 15-day window size.

**Table 3:** Result of daily movement modules by circular statistical analysis. Data are shown in meters (*m*) and outliers are highlighted in bold.

Station	Values	$\overline{mag}$	$\max (mag)$	$\min (mag)$	$\sum {mag}$	Deviation	Kurtosis
ACOR	8829	0.0020	0.0358	0.000000	17.5995	0.0013	59.1373
ALAC	9333	0.0020	0.0132	0.000028	18.4176	0.0013	5.4180
ALBA	7990	0.0017	0.0268	0.000022	13.7388	0.0011	46.5985
ALME	8739	0.0021	0.0242	0.000009	18.4348	0.0015	20.4154
BCLN	4525	0.0022	0.0154	0.000043	9.8263	0.0013	5.3574
CACE	8387	0.0019	0.0155	0.000048	15.9052	0.0013	9.6224
CANT	8763	0.0023	0.0122	0.000011	20.2617	0.0014	3.0889
CEU1	6088	0.0025	0.0206	0.000014	15.1011	0.0019	8.0500
COBA	7134	0.0017	0.0088	0.000024	12.2495	0.0010	2.4333
HUEL	7466	0.0019	0.0169	0.000024	14.4081	0.0014	15.8298
IBIZ	6189	0.0022	**0.3518**	0.000028	13.5611	0.0063	**2193.9395**
IZAN	5897	0.0022	0.0346	0.000025	13.0732	0.0024	45.7510
LEON	6340	0.0018	0.0197	0.000014	11.2938	0.0011	15.9303
LPAL	8408	0.0021	0.0396	0.000015	17.2451	0.0016	63.6651
MALA	8788	0.0020	0.0181	0.000033	17.6464	0.0014	10.4552
MALL	8749	0.0021	0.0228	0.000009	18.7756	0.0015	11.1817
MELI	4533	0.0022	0.0170	0.000029	9.9951	0.0015	9.0173
RIO1	4563	0.0020	0.0138	0.000020	9.0371	0.0013	8.3619
SALA	6519	0.0018	0.0175	0.000020	11.4257	0.0012	17.2307
SONS	8597	0.0018	0.0159	0.000014	15.4470	0.0012	11.3623
TAR0	1841	0.0023	0.0366	0.000050	4.2763	0.0018	88.5349
TERU	5761	0.0018	0.0084	0.000024	10.1879	0.0011	2.4581
VALA	6125	0.0016	0.0137	0.000017	9.7575	0.0010	7.3644
VALE	8529	0.0019	0.0185	0.000024	16.2754	0.0012	10.4100
VIGO	8260	0.0020	0.0162	0.000017	16.8353	0.0014	6.7951
YEBE	9000	0.0018	0.0275	0.000020	16.3955	0.0012	52.7753
ZARA	6646	0.0018	0.0149	0.000014	11.9149	0.0011	7.8294

**Fig. 2:**
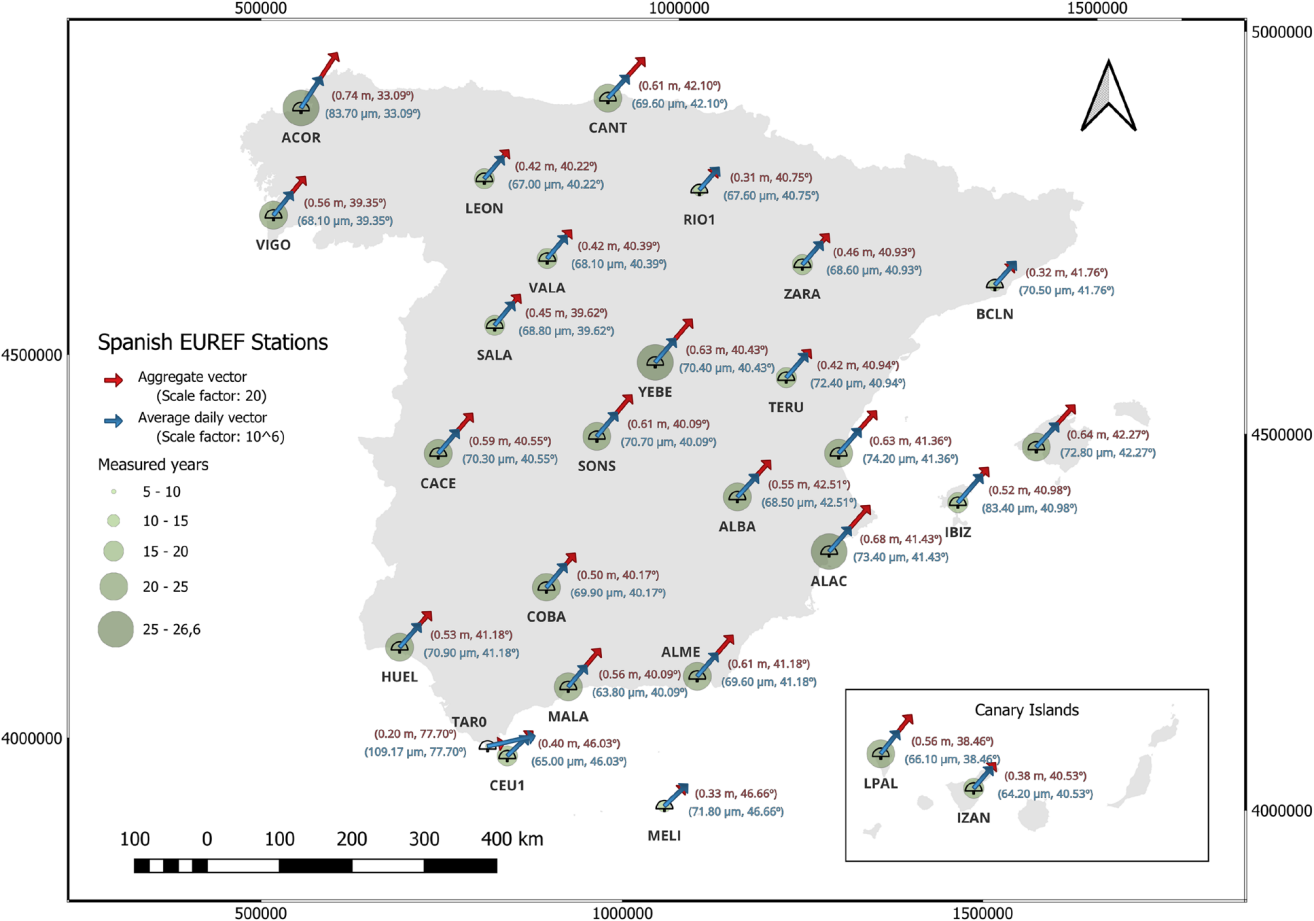
Geographic position of the aggregated and average daily vectors of positional increments of the Spanish EUREF Stations (As the legend indicates: the aggregated vectors are in red at a scale of 20 and the daily vectors in blue at a scale of $10^6$). Map generated with QGIS 3.34 LTR^26^.

**Table 4:** Result of daily displacement angles by circular statistical analysis. Angles are shown in degrees $({}^\circ )$ and outliers are highlighted in bold.

Station	Values	$\overline{\alpha }$	Deviation (*s*)	Dispersal (*D*)	Von-Misses ($\kappa$)	Skewness (*b*)	Kurtosis (*k*)
ACOR	8829	29.23	2.56	337.10	0.08	0.03	0.01
ALAC	9333	33.52	2.59	403.99	0.07	−0.02	−0.01
ALBA	7990	47.73	2.61	455.41	0.07	−0.01	−0.01
ALME	8739	43.05	2.71	740.26	0.05	−0.00	0.03
BCLN	4525	37.20	2.70	674.71	0.05	−0.06	−0.01
CACE	8387	30.49	2.62	450.28	0.06	0.01	0.05
CANT	8763	43.17	2.70	586.77	0.05	0.19	−0.03
CEU1	6088	44.23	2.76	953.32	0.04	−0.06	−0.05
COBA	7134	36.98	2.76	989.38	0.04	−0.02	−0.01
HUEL	7466	32.47	2.57	351.30	0.07	−0.03	0.03
IBIZ	6189	40.46	2.65	525.81	0.06	−0.04	−0.00
IZAN	5897	29.86	2.64	482.60	0.06	−0.05	−0.06
LEON	6340	44.35	2.63	500.14	0.06	−0.00	0.01
LPAL	8408	29.66	2.70	714.98	0.05	0.01	0.03
MALA	8788	26.54	2.65	549.80	0.06	−0.02	0.00
MALL	8749	50.19	2.70	713.28	0.05	−0.04	−0.02
MELI	4533	44.12	2.71	671.00	0.05	−0.11	0.01
RIO1	4563	30.67	2.75	910.49	0.05	0.02	−0.03
SALA	6519	37.82	2.64	509.93	0.06	−0.01	0.01
SONS	8597	50.44	2.57	368.62	0.07	−0.01	0.01
TAR0	**1841**	**70.74**	2.58	369.85	0.07	0.00	−0.05
TERU	5761	41.64	2.69	667.46	0.05	−0.03	0.03
VALA	6125	41.79	2.58	390.54	0.07	−0.02	−0.00
VALE	8529	35.92	2.56	343.22	0.08	−0.02	0.01
VIGO	8260	35.00	2.59	398.12	0.07	0.02	0.04
YEBE	9000	42.56	2.64	522.68	0.06	0.01	−0.00
ZARA	6646	29.31	2.71	769.84	0.05	−0.01	0.02

**Table 5:** Comparison between the velocity vectors published by EUREF and the vectors calculated in this study using strategy 3. Modules are shown in micrometer per day ($\mu m/d$) and angles in degrees $({}^\circ )$.

Station	**Velocity EUREF (m/y)**			**Velocity EUREF**		**Strategy 3**		**Absolute Error**		**Relative Error**	
	$\text {v}_{x}$	$\text {v}_{y}$	$\text {v}_{z}$	Module	Angle	Module	Angle	Module	Angle	Module (%)	Angle (%)
ACOR	−0.01	0.0226	0.0107	73.6680	35.15	83.6676	33.09	9.9996	2.06	13.57	5.85
ALAC	−0.0104	0.02	0.013	71.3042	41.64	73.3788	41.43	2.0746	0.21	2.91	0.50
ALME	−0.0089	0.0192	0.0131	68.1626	41.27	68.5232	42.51	0.3606	1.24	0.53	3.01
ALBA	−0.0148	0.0194	0.0086	68.5252	41.63	69.6096	41.18	1.0844	0.45	1.58	1.07
BCLN	−0.0118	0.0193	0.012	70.2699	43.01	70.5368	41.76	0.2669	1.25	0.38	2.91
CACE	−0.009	0.0194	0.0129	68.4561	40.80	70.3283	40.55	1.8722	0.25	2.73	0.60
CANT	−0.0106	0.0191	0.0118	67.9835	41.76	69.6463	42.1	1.6628	0.34	2.45	0.80
CEU1	−0.0089	0.0167	0.0139	64.3326	46.33	65.0366	46.03	0.7040	0.30	1.09	0.66
COBA	−0.0093	0.0194	0.0125	68.1157	40.63	69.9484	40.17	1.8327	0.46	2.69	1.14
HUEL	−0.0086	0.0191	0.0137	68.6400	41.88	70.9094	41.18	2.2694	0.70	3.31	1.68
IBIZ	−0.0117	0.0198	0.0122	71.3702	42.24	83.4034	40.98	12.0332	1.26	16.86	2.98
IZAN	−0.0052	0.0175	0.0138	63.7223	41.29	64.2432	40.53	0.5209	0.76	0.82	1.85
LEON	−0.0097	0.0188	0.0122	66.9064	41.57	66.9722	40.22	0.0658	1.35	0.10	3.25
LPAL	−0.004	0.018	0.0147	66.1982	40.25	66.0951	38.46	0.1031	1.79	0.16	4.45
MALA	−0.0081	0.0178	0.0129	64.1694	42.19	63.7735	40.09	0.3959	2.10	0.62	4.98
MALL	−0.0119	0.0199	0.0123	72.0659	42.48	72.8228	42.27	0.7569	0.21	1.05	0.49
MELI	−0.0108	0.0181	0.0151	70.9682	47.49	71.8157	46.66	0.8475	0.83	1.19	1.75
RIO1	−0.0104	0.0191	0.0118	67.7543	41.49	67.5782	40.75	0.1761	0.74	0.26	1.79
SALA	−0.0097	0.0195	0.0125	68.7849	40.96	68.8292	39.62	0.0443	1.34	0.06	3.27
SONS	−0.0096	0.0194	0.0124	68.3006	40.85	70.7352	40.09	2.4346	0.76	3.56	1.86
TAR0	−0.0119	0.0152	0.0155	67.6927	54.04	109.1703	77.7	41.4776	23.66	61.27	43.78
TERU	−0.0101	0.0199	0.011	68.1070	38.82	72.4289	40.94	4.3219	2.12	6.35	5.45
VALA	−0.0091	0.0193	0.0127	68.6321	41.48	68.1391	40.39	0.4930	1.09	0.72	2.64
VALE	−0.0112	0.0194	0.0121	69.7117	42.23	74.2437	41.36	4.5320	0.87	6.50	2.06
VIGO	−0.0092	0.0193	0.0119	67.1524	39.84	68.0653	39.35	0.9129	0.49	1.36	1.23
YEBE	−0.0091	0.0194	0.0125	68.5481	41.10	70.4128	40.43	1.8647	0.67	2.72	1.62
ZARA	−0.0111	0.0193	0.0117	68.8726	41.87	68.5728	40.93	0.2998	0.94	0.44	2.25

**Fig. 3:**
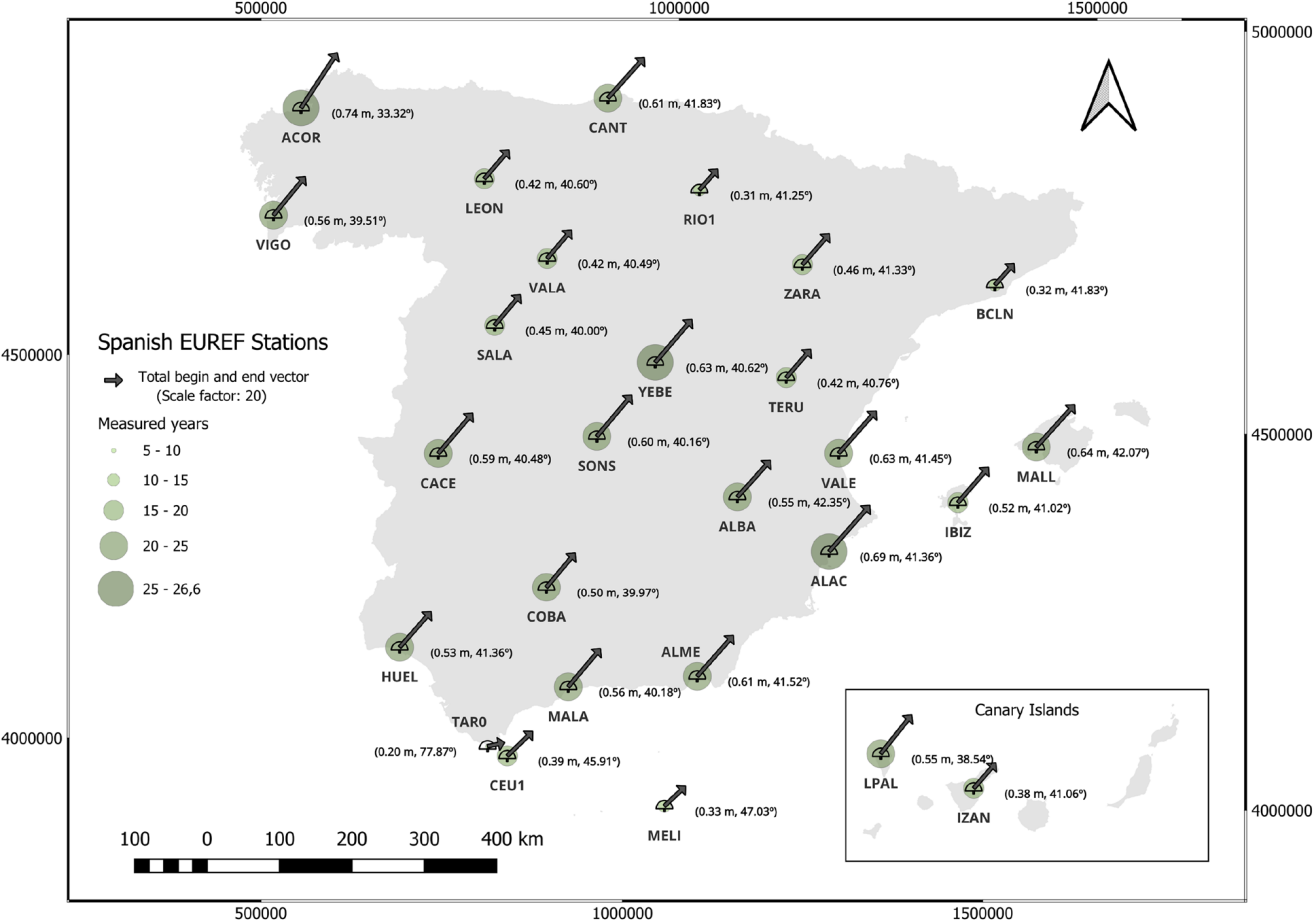
Geographic position of the total start and end average daily vector of positional increments of the Spanish EUREF Stations. (As the legend indicates: the vectors are at a scale of $10^6$). Map generated with QGIS 3.34 LTR^26^.

Tables 3 and 4 provide the circular statistical analysis of the movements for each station, both in terms of magnitude (*mag*) (Table 3) and angle $(\alpha )$ (Table 4). The statistical results presented in Tables 3 and 4 are derived from the analysis of magnitudes and angles obtained from the daily displacements. It is worth noting that the final movement of a station is around 10 meters, as this magnitude varies daily and does not reflect a trend or statistical significance. In contrast, the displacement of the stations does provide statistical significance, as it shows the variation in the station’s position over the course of the historical data series.

Figures 2 and 3 illustrate the displacements obtained using the three defined strategies. Figure 2 corresponds to Strategies 1 and 3, showing the daily displacement vectors for all analyzed permanent stations. Blue vectors, scaled to $10^6$, depict the average daily movement. In contrast, the red vectors, scaled at 1:20, show the aggregated displacement from the study. The number of years of the study is indicated by green circles. Figure 3 presents the results of strategy 2, showing the displacement vectors calculated as the difference between the means of the first 15 positions of the station and the last 15 measured positions, represented in black and scaled to 1:20.

**Fig. 4:**
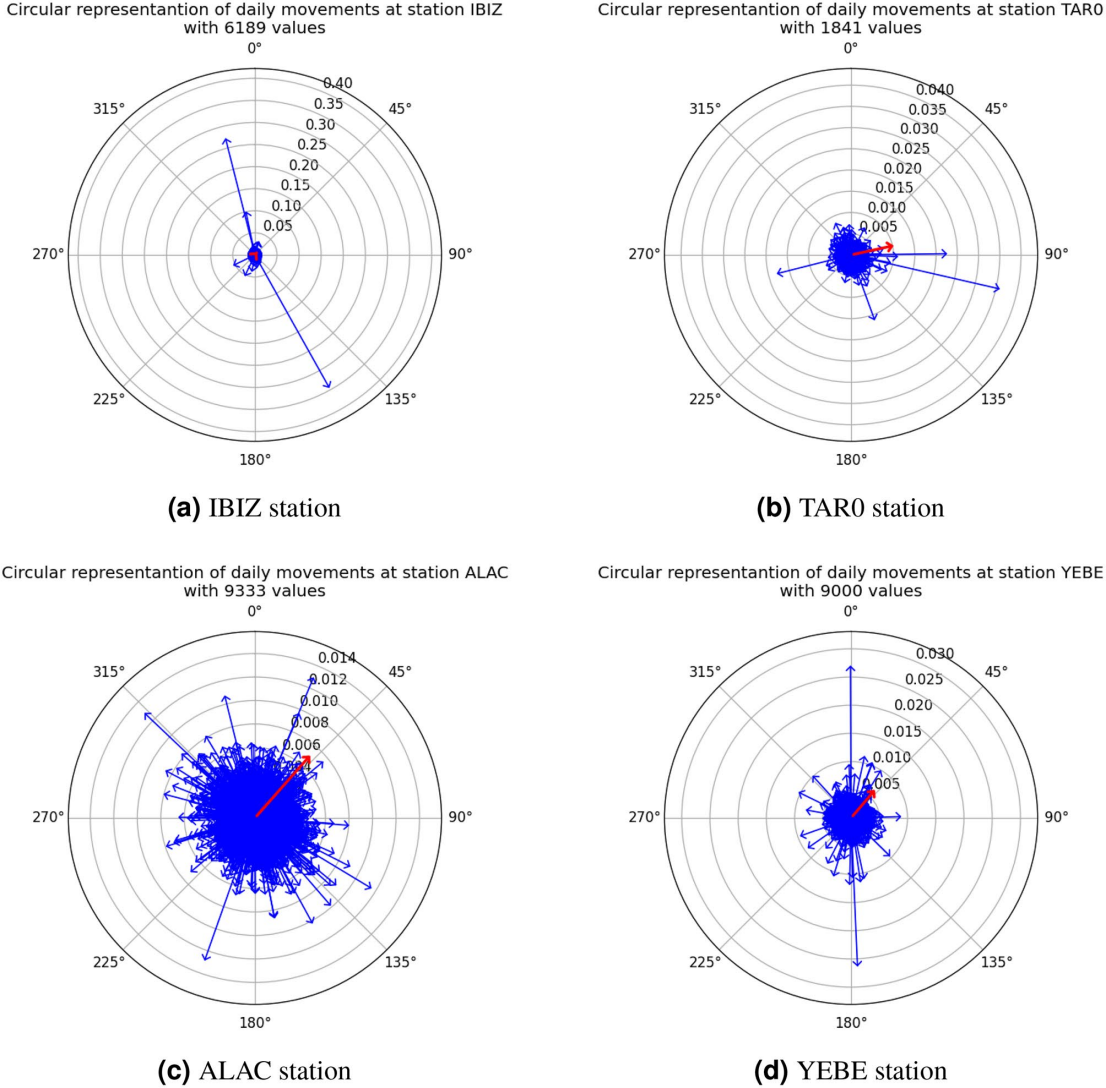
Circular representation of daily displacement at some stations. The red arrow represents the average displacement over the historical series.

The study reveals that all permanent stations experience a northeastward displacement of approximately 1 mm every 14 days. The displacement magnitudes are very similar across the analyzed stations, with average values around 2 mm and maximum values ranging between 2 and 3 cm. An exception is observed at the IBIZ station, which exhibits a significantly higher maximum displacement of 35 cm, as well as a markedly elevated kurtosis compared to the other stations. The results regarding the angular trend of the displacements range from $38.46^{\circ }$ to $46.66^{\circ }$, except for the TAR0 station, which shows a distinctly different orientation of $77.7^{\circ }$. The circular representation of the measured results at the IBIZ station can be seen in Fig. 4a, while those corresponding to the TAR0 station are shown in Fig. 4b. Additionally, the circular representations for the permanent stations with the highest number of measured values (ALAC and YEBE) are presented in Fig. 4c and d, respectively. In these figures, each blue arrow represents the magnitude and angle of daily movement, while the red arrow represents the average displacement over the historical series. The magnitude of the average displacement is shown at a scale of 100:1.

In Figure 4a, it can be observed that most displacements are highly centralized; however, three vectors stand out with magnitudes ranging from 10 to 35 cm, which explains the high variation in Kurtosis. Nevertheless, since a total of 6,189 measurements are available, these three displacements do not significantly affect the mean displacement, which maintains the same direction and magnitude as the other stations. These large variations occur in three intervals during which the station ceased to operate, ranging from two months to a year and a half, depending on the specific displacement. This may be due to the need for station maintenance, the recalculation of its position, or construction work on the hosting building. On the other hand, in Fig. 4b, it can be observed that the TAR0 station presents two vectors with large magnitude oriented towards the north. In this case, and in contrast to the IBIZ station, the lower number of available measurements has a significant impact on the result, making it the only station with a displacement angle above $50^\circ$. This may be due to the limited number of valid observations, since those recorded prior to June 6, 2019 are invalid as a result of a substantial displacement of 8 meters, caused by a relocation of the station. Figures 4c and d illustrate examples of stations that have been continuously recording measurements, resulting in a wide variety of data. Although some records exhibit large variations (greater than 10 cm per day), these are offset by the consistent daily observations, leading to a stable northeastward trend across the entire national territory.

To verify the validity of the study, the calculated daily displacements are compared with the velocity vectors published by EUREF. The agency periodically publishes the precise position of the station ($x(t_0)$) on a specific date or initial time ($t_0$), together with the observed velocity (*v*). From there, the new position can be obtained by a linear combination between the initial position and the observed time (*t*).

10 $$\begin{aligned} x(t) = x(t_0) + (t - t_0) \cdot v \end{aligned}$$

EUREF publishes these data in the ETRF2000 (EPSG:7930), ETRF2014 (EPSG:8401) and IGb14 (EPSG:9378) representation systems. The three representation systems show positions using geocentric 3D Cartesian coordinates, while this study was carried out in the UTM30N/WGS84 (EPSG:32630) representation system, which uses a 2D Cartesian coordinate system. The conversion of the velocity vectors themselves between a 3D representation system and another projected system cannot be performed directly. To obtain these data, the linear combination of Equation 10 is used to calculate the velocity. For each station, the position is obtained for the instant $t_0$ and $t_0+1$; both positions are projected in UTM30N from IGb14, and the difference is calculated. This difference between the final and initial positions corresponds to the daily displacement for each station projected in UTM30N. Table 5 shows the velocity vector published by EUREF for each station in the IGb14 representation system, presented as annual displacement (m/y) in geometric coordinates. The representation of this velocity vector is projected in UTM30N as modulus and angle which are subsequently converted to daily displacements ($\mu$m/d) in polar coordinates, as well as the modulus and angle calculated in this study for the third strategy, which represents the observed daily displacement at that station. The last columns show the absolute and relative errors between both vectors.

Table 5 shows that the median relative error for the modules is $1.36\%$, while for the angles it is around $1.86\%$. These errors translate into median absolute errors of $0.91\mu m /d$ for the module and $0.83^\circ$ for the direction. If these data are extrapolated to the error calculated on an annual basis, a difference of 0.33*mm*/*y* is obtained, with a median displacement of 2.53*cm*/*y*, assuming a relative error of approximately $1\%$. Even so, it is worth noting the three outliers obtained for the ACOR, IBIZ and TAR0 stations. According to the EUREF register (https://www.epncb.oma.be/ftp/station/coord/EPN/EPN_discontinuities.snx), these stations have experienced different periods of inactivity as well as various maintenance issues, which means that the number of values measured does not correspond on a daily basis, and their daily average standard is affected. Excluding these values, the data certify that the error observed between the data calculated in this study and the data published by EUREF show minimal differences, most of which are due to projection errors and small deviations from some stations.

Discontinuity periods have been accounted for by adjusting the number of observation values (N) used in the calculations. The daily displacement is computed by dividing the cumulative increments by N (the number of available measurements) rather than by the total number of calendar days. Consequently, stations with more frequent data gaps exhibit larger differences between strategies, as N diverges increasingly from the total day count. Additionally, minor numerical discrepancies may arise from floating-point representation limitations (numpy.float64 precision), particularly during coordinate transformations involving numbers with highly significant decimal components. If any library does not maintain adequate precision, these values may be slightly altered, contributing to small discrepancies between EUREF-published velocities and those computed in this study.

Quality control was performed using IGN station logs, the same files used to verify the stability of the 15-day windows. Stations with extended initial instabilities (e.g., IBIZ, $\approx$1 year unstable data) were analyzed only from the stabilized period onward. When data gaps occurred, position increments were calculated between consecutive available epochs, and missing days were excluded from the observation count (N), thus maintaining consistency between cumulative displacement and the number of actual measurements.

Finally, Table 6 presents a comparison between the velocity product provided by EUREF and the secular velocity calculated in this study. Specifically, the secular velocity vectors are compared in the IGb14 reference frame. To perform this comparison, EUREF data are maintained as published, while the data from this study are transformed. For this transformation, the initial position ($p_1$) of each station is incremented by the calculated secular displacement vector, obtaining the final position ($p_2$). Both positions in UTM30N (EPSG:32630) are transformed to IGb14 (EPSG:9378), and the velocity vector is calculated considering the number of days elapsed between the first and last observation. With these two vectors –the first denoted as Velocity EUREF (m/y) and the second as Study’s Velocity (m/y)– two types of errors are computed: the relative error between the magnitudes of the two vectors, and the angle between them in the plane they define. Only 3 out of 27 stations show an relative error greater than 5% in the secular velocity vector magnitude, and 4 stations present an angle between the two vectors greater than $5^\circ {}$. Therefore, both vectors exhibit a high degree of similarity for the majority of stations. The exceptional case of station TAR0, which presents a significantly larger error (52.42% and $23.95^\circ {}$), is attributed to its station relocation in June 2019, as discussed in detail in Fig. 4b.

**Table 6:** Velocity vector comparison between EUREF and this study in the IGb14 reference frame.

Station	**Velocity EUREF (m/y)**				**Study’s Velocity (m/y)**				**Error**	
	$\text {v}_{x}$	$\text {v}_{y}$	$\text {v}_{z}$	Module	$\text {v}_{\Delta {x}}$	$\text {v}_{\Delta {y}}$	$\text {v}_{\Delta {z}}$	Module	Module (%)	Angle ($\phantom{0}^\circ$)
ACOR	−0.0100	0.0226	0.0107	0.0269	−0.0083	0.0243	0.0124	0.0286	6.32	5.21
ALAC	−0.0104	0.0200	0.0130	0.0260	−0.0101	0.0199	0.0129	0.0258	0.77	0.54
ALME	−0.0089	0.0192	0.0131	0.0249	−0.0088	0.0189	0.0128	0.0245	1.61	0.21
ALBA	−0.0148	0.0194	0.0086	0.0259	−0.0097	0.0187	0.0128	0.0246	5.02	14.76
BCLN	−0.0118	0.0193	0.0120	0.0256	−0.0110	0.0194	0.0117	0.0252	1.56	1.54
CACE	−0.0090	0.0194	0.0129	0.0250	−0.0086	0.0193	0.0129	0.0247	1.20	0.86
CANT	−0.0106	0.0191	0.0118	0.0248	−0.0103	0.0190	0.0122	0.0249	0.40	1.15
CEU1	−0.0089	0.0167	0.0139	0.0235	−0.0085	0.0165	0.0138	0.0231	1.70	0.75
COBA	−0.0093	0.0194	0.0125	0.0249	−0.0083	0.0191	0.0127	0.0244	2.01	2.10
HUEL	−0.0086	0.0191	0.0137	0.0250	−0.0080	0.0190	0.0135	0.0246	1.60	1.06
IBIZ	−0.0117	0.0198	0.0122	0.0260	−0.0105	0.0200	0.0124	0.0258	0.77	2.63
IZAN	−0.0052	0.0175	0.0138	0.0229	−0.0032	0.0170	0.0146	0.0226	1.31	5.62
LEON	−0.0097	0.0188	0.0122	0.0244	−0.0091	0.0190	0.0119	0.0242	0.82	1.44
LPAL	−0.0040	0.0180	0.0147	0.0236	−0.0026	0.0178	0.0146	0.0232	1.69	3.24
MALA	−0.0081	0.0178	0.0129	0.0234	−0.0076	0.0180	0.0121	0.0230	1.71	2.13
MALL	−0.0119	0.0199	0.0123	0.0262	−0.0113	0.0198	0.0126	0.0261	0.38	1.39
MELI	−0.0108	0.0181	0.0151	0.0259	−0.0097	0.0179	0.0150	0.0253	2.32	2.04
RIO1	−0.0104	0.0191	0.0118	0.0247	−0.0099	0.0190	0.0116	0.0244	1.21	1.01
SALA	−0.0097	0.0195	0.0125	0.0251	−0.0088	0.0194	0.0122	0.0246	1.99	1.78
SONS	−0.0096	0.0194	0.0124	0.0249	−0.0093	0.0200	0.0128	0.0255	2.41	1.35
TAR0	−0.0119	0.0152	0.0155	0.0248	−0.0210	0.0092	0.0300	0.0378	52.42	23.95
TERU	−0.0101	0.0199	0.0110	0.0249	−0.0100	0.0196	0.0123	0.0252	1.20	2.91
VALA	−0.0097	0.0193	0.0127	0.0251	−0.0093	0.0193	0.0122	0.0247	1.59	1.09
VALE	−0.0112	0.0194	0.0121	0.0255	−0.0103	0.0198	0.0126	0.0256	0.39	2.50
VIGO	−0.0092	0.0193	0.0119	0.0245	−0.0084	0.0192	0.0124	0.0243	0.82	2.25
YEBE	−0.0097	0.0194	0.0125	0.0250	−0.0095	0.0196	0.0123	0.0250	0.00	0.67
ZARA	−0.0111	0.0193	0.0117	0.0252	−0.0101	0.0192	0.0117	0.0247	1.98	1.94

The results show that all three strategies are valid for calculating tectonic plate movements by measuring the precise position of permanent stations. Firstly, strategies 1 and 2 seek to determine the displacement generated from the installation of the receiver to the end date of this study, while strategy 3 aims to predict the daily and annual displacement of the stations and, consequently, of the Eurasian tectonic plate. Thus, strategies 1 and 2 are useful for determining cumulative displacement over long periods and calculating secular velocity with greater precision, while strategy 3 is relevant for characterizing daily displacement rates. The average displacements obtained in this study are $69.27\mu m/d$ (2.53*cm*/*y*) with an angle of $41.21^\circ$, while the average data calculated with EUREF velocities correspond to $68.15\mu m/d$ (2.49*cm*/*y*) with an angle of $41.78^\circ$, consisting of an annual displacement of the Eurasian tectonic plate of approximately 2.5 cm in a north-easterly direction. These average data do not include the ACOR, IBIZ and TAR0 stations, as they are considered outliers.

## Conclusions

The geodetic networks of Spain have multiple missions, among which the following are highlighted: 1) unifying the Reference System at the national level; and 2) improving the accuracy of geodetic and topographic projects by increasing the speed of data collection. The case study presented in this article was developed by analysing several permanent stations using circular statistics. Based on the circular statistical analysis of the data generated from the station coordinates, it is concluded that the movements observed are very small (on the order of micrometres per day). Furthermore, all parameters of the mean direction of colatitude and the mean direction of longitude are within the same quadrant. In contrast, daily movements show considerable variability and, over extended periods, can occur in multiple directions. However, a clear directional trend is observed at all stations which, when a set of measurements is available, converge in a trend movement. The displacement trend of the Eurasian tectonic plate in Spain is of the order of 2.53 cm per year in a north-easterly direction, with an angle of $41.21^\circ$. The direction of these vectors is consistent with the direction of the tectonic movement of the Eurasian plate^35^.

## Data Availability

The data used as precise daily positions for each of the stations in the EUREF network on Spanish territory were obtained from the Nevada Geodetic Laboratory (https://geodesy.unr.edu/). This data acquisition was carried out with the support of the Instituto Geográfico Nacional (https://www.ign.es/) of Spain, which also provided the station metadata.
